# Evaluation of acupuncture and auriculotherapy in the control of chemotherapy-induced nausea and vomiting: a Pilot Study*

**DOI:** 10.1590/1980-220X-REEUSP-2023-0191en

**Published:** 2023-10-30

**Authors:** Sabrina Ferreira Monteiro Morais, Ruth Natalia Teresa Turrini

**Affiliations:** 1 Universidade de São Paulo, Escola de Enfermagem, Departamento de Enfermagem Médico-Cirúrgica, São Paulo, SP, Brazil.

**Keywords:** Acupuncture, Antineoplastic Agents, Nausea, Vomiting, Nursing, Acupuntura, Antineoplásicos, Náusea, Vómitos, Enfermería, Acupuntura, Antineoplásicos, Náusea, Vômito, Enfermagem

## Abstract

**Objective:**

To evaluate the effectiveness of acupuncture and auriculotherapy protocol in relieving chemotherapy-induced nausea and vomiting in cancer patients compared to the antiemetic protocol.

**Method:**

Pilot study of a pragmatic two-arm clinical trial: an acupuncture group received systemic acupuncture, auriculotherapy, and antiemetic protocol; a control group used antiemetic protocol. The sample consisted of 42 patients with cancer of the gastrointestinal system or multiple myeloma. The outcome was assessed using the Chemotherapy-Induced Nausea and Vomiting Assessment Tool and the patient’s diary.

**Results:**

There was no statistically significant difference between groups according to the assessment of the patient’s diary and the Assessment Tool of chemotherapy-induced nausea and vomiting. The patients were 60 years old on average and the groups were homogeneous, except for marital status. In the diary, there was no statistical difference between groups and sessions for days of nausea (p = 0.873) and vomiting episodes (p = 0.993).

**Conclusion:**

The protocol of acupuncture and auriculotherapy as a complementary treatment of chemotherapy-induced nausea and vomiting was ineffective, considering the limitations of the study.

## RESUMO

**Objetivo:**

Avaliar a efetividade do protocolo de acupuntura e auriculoterapia no alívio de náuseas e vômitos induzidos por quimioterapia em pacientes oncológicos em comparação ao protocolo antiemético.

**Método:**

Estudo piloto tipo ensaio clínico pragmático de dois braços: grupo acupuntura recebeu acupuntura sistêmica, auriculoterapia e protocolo antiemético, o grupo controle utilizou protocolo antiemético. A amostra foi composta por 42 pacientes com câncer do sistema gastrointestinal ou mieloma múltiplo. O desfecho foi avaliado pelo Instrumento de Avaliação de náuseas e vômitos induzidos por quimioterapia e diário do paciente.

**Resultados:**

Não houve diferença estatística significativa entre os grupos pela avaliação do diário do paciente e Instrumento de Avaliação de náuseas e vômitos induzidos por quimioterapia. Os pacientes apresentavam em média 60 anos e os grupos foram homogêneos, exceto para o estado civil. No diário, não houve diferença estatística entre os grupos e as sessões para os dias de náuseas (p = 0,873) e episódios de vômitos (p = 0,993).

**Conclusão:**

O protocolo de acupuntura e auriculoterapia como tratamento complementar de náuseas e vômitos induzidos por quimioterapia foi inefetivo, considerando as limitações do estudo.

## RESUMEN

**Objetivo:**

Evaluar la efectividad del protocolo de acupuntura y auriculoterapia para aliviar las náuseas y los vómitos inducidos por la quimioterapia en pacientes con cáncer en comparación con el protocolo antiemético.

**Método:**

Estudio piloto tipo ensayo clínico pragmático con dos brazos: el grupo de acupuntura recibió acupuntura sistémica, auriculoterapia y protocolo antiemético, el grupo control utilizó un protocolo antiemético. La muestra estuvo compuesta por 42 pacientes con cáncer del sistema gastrointestinal o mieloma múltiple. El resultado se evaluó mediante el Instrumento de Evaluación de Náuseas y Vómitos inducidos por quimioterapia y el diario del paciente.

**Resultados:**

No hubo diferencias estadísticamente significativas entre los grupos según la evaluación del diario del paciente y el Instrumento de Evaluación de náuseas y vómitos inducidos por quimioterapia. Los pacientes tenían en promedio 60 años y los grupos eran homogéneos, excepto por el estado civil. En el diario no hubo diferencia estadística entre los grupos y sesiones para los días de náuseas (p = 0,873) y episodios de vómitos (p = 0,993).

**Conclusión:**

El protocolo de acupuntura y auriculoterapia como tratamiento complementario de las náuseas y vómitos inducidos por la quimioterapia resultó ineficaz, considerando las limitaciones del estudio.

## INTRODUCTION

Traditional, Complementary and Integrative Medicines (TCIM), as named by the World Health Organization, refer to the set of health care practices based on theories about illness processes from different cultures used for health promotion, recovery, and prevention, which approaches the individual in an integral way in his/her biopsychosocio-spiritual dimensions^(1)^. In Brazil, TCIMs are known as Integrative and Complementary Health Practices (*Práticas Integrativas e Complementares em Saúde* - *PICS*) and their use in the Brazilian Public Health System (*SUS*) has expanded primary health care^(2)^. However, for cancer, which requires highly complex care, integrative oncology has been adopted especially in hospitals^(3)^.

The Traditional Chinese Medicine (TCM) has a set of traditional practices, developed over more than 5,000 years. Among them, acupuncture aims at health promotion and recovery in its entirety, used alone or in conjunction with other therapies. This therapy consists of inserting thin needles deep into the skin at specific anatomical sites, known as acupuncture points, aimed at preventing and recovering health problems^(2)^. Another TCM practice is auriculotherapy which, by stimulating the pinna, is used to relieve pathological situations in the body, and has two main lines of reasoning to explain its principles, the Chinese school (TCM) and the French school (Paul Nogier)^(4)^.

Acupuncture as an integrative treatment in the management of toxic effects induced by chemotherapy (CT) is one of the great contributions of TCM for cancer patients, as it can help increase the patient’s tolerance to the CT protocol and favor the continuation of the CT planning without interruptions, providing innovative care^(5)^.

Cancer is one of the main public health problems in the world and represents one of the main causes of premature death (before than 70 years old) in most countries. The number of cases of cancer and mortality increase each year due to population growth, in part due to aging, and to the distribution and prevalence of multiple risk factors for cancer, some related to socioeconomic development^(6)^.

In the present study, the focus will be on patients with cancer of the gastrointestinal system and Multiple Myeloma (MM), a solid and a hematological cancer, respectively, both malignant. Among the main therapeutic modalities, there is CT, which is the systemic form that uses drugs called chemotherapeutics or antineoplastics, administered at regular intervals depending on the therapeutic regimen^(7)^. Each type of cancer has a chemotherapy protocol. In this study, patients with gastrointestinal system cancer used the protocol with Oxaliplatin, Leucovorin and 5-Fluorouracil (BFOL) and those with MM, the protocol with Cyclophosphamide, Bortezomib, and Zoledronic Acid (CYBORD).

As the adverse events commonly reported in the use of antineoplastic drugs are chemotherapy-induced nausea and vomiting (CINV), these drugs are classified according to their emetogenic potential^(8)^, and the one with the greatest emetogenic potential characterizes the protocol. Therefore, Cyclophosphamide is a drug with a high emetogenic potential and Oxaliplatin has a moderate emetogenic potential^(9)^.

CINVs occur due to a direct action of antioneoplasic drugs on the central nervous system, where the trigger zone of the medulla detects the presence of “foreign” substances in the body and releases stimuli to the vomiting center in the medulla, which, in its turn, disperses efferent stimuli to different regions of the body, triggering vomiting. There may also be direct stimulation of the gastrointestinal tract through the individual’s memory and learning mechanisms, which would explain the episodes of anticipatory vomiting that may occur before the CT session, in later cycles of treatment^(10)^. Five different types of CINV have been described: acute, late, incidental, anticipatory, and refractory^(11)^.

In the TCM, CT affects Wood (Liver and Gallbladder) and Earth (Spleen and Stomach) elements. The Stomach sends processed food down to the Small Intestine, so in terms of health, the Qi (vital energy) of the Stomach has a downward movement. In the disease, the stomach is affected by stagnation of Qi, rebellion of Qi (ascent rather than descent of Qi) or retention of food, leading to a feeling of fullness and distention, sour regurgitation, belching, hiccups, nausea and vomiting, which are caused by Dampness obstructing the Middle Burner and preventing Stomach-Qi from descending^(12)^. Considering these pathophysiological aspects, acupuncture has been used in cancer patients to relieve nausea, vomiting, pain, fatigue, xerostomia, and other chemotherapy adverse effects.

TCM postulates the theory that stimulation of the PC6 acupuncture point regulates Stomach Qi function and subsequently prevents nausea and vomiting^(13)^. An observational study explored the effectiveness of acupuncture combined with antiemetic drugs in preventing and treating CINV in patients with breast cancer using postoperative adjuvant chemotherapy (anthracycline and cyclophosphamide), comparing a control group (CG) with an antiemetic protocol and an acupuncture group with an antiemetic regimen combined with acupuncture. Over the five days of the study, the number of patients without CINV or post-CT vomiting or nausea only increased, and the number with CINV or vomiting only decreased in the acupuncture group (p = 0.046)^(14)^.

Studies on the use of TCM to control nausea and vomiting have varied methodologies, mainly in relation to the technique for stimulating the acupoint, the intervention protocol, and the number of points used, but the PC6 acupoint for the treatment of nausea and vomiting of different etiologies was unanimously used. This study aimed to evaluate the effectiveness of the acupuncture and auriculotherapy protocol in relieving CINV in cancer patients compared to standard antiemetic treatment.

## METHOD

### Design and Place of Study

Pilot study of pragmatic two-arm clinical trial: control group (CG) and systemic acupuncture + auriculotherapy group (GACA), carried out at the Oncology Outpatient Clinic of the Hospital Regional do Vale do Paraíba (HRVP), Taubaté/SP.

### Data Collection

Study developed from May to September 2022.

### Sample/Population

As this is a pilot study, a non-probabilistic convenience sample of 50 participants was established. Patients aged ≥ 18 years with a diagnosis of gastrointestinal cancer or MM, undergoing chemotherapy with high and moderate emetogenic drugs, at the beginning of the chemotherapy cycle were included. The following patients were excluded: those on anticoagulants, antiplatelet agents, with conditions that stimulate nausea and vomiting, such as intestinal obstruction, anorexia, gastrointestinal diseases, non-oncological diseases; with vomiting of central origin (brain metastasis, intracranial hypertension and others); patients with labyrinthopathy, without clinical conditions (fever, infection, severe anemia) to receive acupuncture treatment; patients undergoing chemotherapy with 15-day, 21-day or monthly sessions; patients who received prior acupuncture treatment for CINV; those using medicinal plant, herbal medicine or homeopathy for CINV; needle-fear patients. The distribution of participants in the groups was carried out by drawing lots after randomization 1:1 by the *Research Randomizer* (https://www.randomizer.org/).

### Intervention

GACA received systemic acupuncture with 25 × 30mm needles at the PC6 (*Neiguam*), SP6 (*sanyinjiao*), ST36 (*Zusanli*), RN12 (*zhongwan*), LR14 (*Qimen*) points and auriculotherapy with radionic crystals in the Shenmen, Spleen, Stomach, Anxiety points, Point Zero and Point 29a of motion sickness/nausea. In addition to these pre-defined points, an energy assessment was carried out using the Ryodoraku System, making the service partially individualized. Thus, some patients may have included lung, heart, large intestine, small intestine, gallbladder, and kidney points in their auriculotherapy, and in systemic acupuncture, points LU9, HT7, LI4, SI4, GB40 and KI3. The systemic acupuncture procedure lasted an average of 30 minutes. The radionic crystals were placed at the identified points and fixed with hypoallergenic porous adhesive tape that were kept for one week, and did not require stimulation, alternating the ears at each session. Patients were instructed to remain with the crystals until the next session, after which they were removed by the researcher. Systemic acupuncture and auriculotherapy were applied once a week, regardless of the CT protocol, moments before the CT session.

Both groups used drugs prescribed according to the CT protocol and antiemetics. The CG only used the drugs prescribed according to the protocol.

### Outcomes

The outcome was assessed by the presence of nausea; presence of vomiting; number of days with nausea and vomiting; proportion of treatment days with nausea and vomiting; acute and late vomiting with information obtained from the Patient Diary and the CINV Assessment Tool; assessment of gastrointestinal adverse reactions (nausea and vomiting), as defined by the Guide for Reporting Adverse Reactions in Oncology^(15)^.

### Instruments

The following instruments were used: biosociodemographic questionnaire (sociodemographic and occupational data, data on cancer and comorbidities); CINV questionnaire (occurrence of CINV, antiemetic use, CT and cycle data); proposed home diary for recording CINV and use of antiemetics and teas, between sessions; Guide for Reporting Adverse Reactions in Oncology, based on the translation of the *Common Terminology Criteria for Adverse Events* (CTCAE) – version 4.0, was used to evaluate adverse events and their intensity regarding the toxicity of the CT treatment. The severity of adverse events according to the CTCAE was defined as: GRADE 1 – mild, GRADE 2 – moderate, GRADE 3 – high, GRADE 4 – life-threatening consequences, GRADE 5 – death related to the adverse event^(15)^.

### Recruitment

The patients’ medical records were selected after consultation with the oncologist to define the CT protocol. Patients with high and moderate emetogenic chemotherapy were invited to participate in the research on the day of the consultation to schedule the CT and those who accepted completed the biosociodemographic form to identify the patients eligible for the study and signed the Free Informed Consent Form (FICF). On the day of the first CT session, the patient received a sequential envelope with the information on group of allocation: GACA or CG.

### Blinding

There was no blinding of participants and researcher. The statistician had access to information on group assignment at the end of the study.

### Data Collection Procedure

Patients in all CT sessions completed the CINV questionnaire after the session and were instructed to complete the nausea and vomiting diary at home until the next session.

### Statistical Analysis

Patients’ characterization was presented through relative and absolute frequencies, measures of central tendency and variability. The homogeneity of the groups was verified using the chi-square test or Fisher’s exact test for qualitative variables and the Wilcoxon Mann-Whitney test for quantitative variables. Outcomes between groups were also assessed using the Wilcoxon Mann-Whitney test. A generalized linear mixed-effects model was used to compare groups across sessions and differences between protocols. The analysis was performed by a statistician using the R® 4.0.4 software and a significance level of 5%.

### Ethical Aspects

The project was approved by the Research Ethics Committee of the Hospital Regional do Vale do Paraíba, of the Universidade de Taubaté (opinion no. 5.343.476), approved on 04/11/2022, and of the Nursing School of the Universidade de São Paulo (opinion no. 5.266.059), approved on 02/25/2022, in accordance with the recommendations of the Brazilian National Committee for Research Ethics (CONEP Resolution 466/2012. The participants signed the FICF and those from the CG who wished, at the end of the study, received acupuncture and Chinese auriculotherapy.

## RESULTS

**Figure 1:**
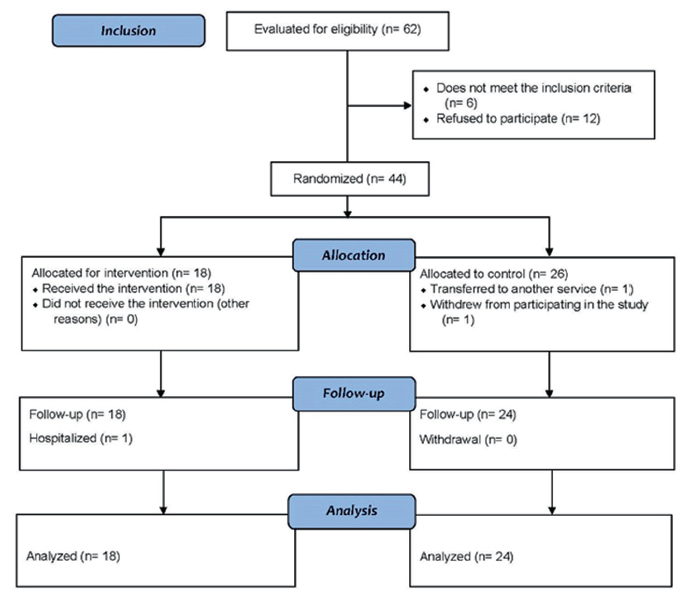
Clinical trial flowchart according to CONSORT. Sao Paulo, 2022.

The total number of medical records of selected patients was 62 and 12 (16.2%) did not accept to participate in the study. Six (8.8%) patients who were ineligible due to labyrinthopathy (n = 1), cognitive deficit (n = 1), change in the CT protocol (n = 2) and without clinical conditions to receive treatment with acupuncture (n=1) were excluded. Two eligible patients quit before the first meeting and there were losses throughout the study (Figure 1).

### Biosociodemographic Characteristics

The sample consisted mainly of female patients (52.4%; n = 22), mostly retired (52.4%; n = 22) or beneficiaries of social security (INSS) or of the Organic Law of Social Assistance - LOAS (19%; n = 8). Most patients were white (61.9%; n = 26), predominantly Catholic (78.6%; n=33), married (54.8%; n = 23), 40.5% (n = 17) had completed high school, and 38.1% (n = 16) had incomplete elementary school. Distribution by study groups showed that they were homogeneous, except for marital status (p = 0.036) (Table 1).

**Table 1:** Distribution of cancer patients according to sociodemographic variables, study group, and p-value – Taubaté, Brazil, May/2022 to Sep/2022.

Variable	Level	GACA		CG		p-value
		N	%	N	%	
Sex	Female	9	40.9	13	59.1	0.792**
	Male	9	45.0	11	55.0	
	Married	6	26.1	17	73.9	
Marital status	Single	6	75.0	2	25.0	0.036*
	Divorced/separated/widow	6	54.6	5	45.4	
	Elementary School	8	40.0	12	60.0	
Level of Education^1^	High school	8	44.4	10	55.6	0.724**
	Higher education	2	50.0	2	50.0	
	White	10	38.5	16	61.5	
Race	Black	4	57.1	3	42.9	0.468**
	Brown	4	44.4	5	55.6	
Occupation	Retired	9	40.9	13	59.1	
	Self Employed/Employee	3	60.0	2	40.0	
	Unemployed	3	42.9	4	57.1	0.791**
	INSS/LOAS^2^	3	37.5	5	62.5	
Religion	Catholic	15	45.4	18	54.6	
	Evangelical	3	37.5	5	62.5	0.519**
	None	–	–	1	100.0	
**Total**		**18**	**42.9**	**24**	**57.1**	

^*^Fisher test;

^**^chi-square test;

^1^each category includes completed or uncompleted education;

^2^LOAS: assistance benefit for socially vulnerable individuals.

The mean age of the patients was around 60 years old, they lived on average 40–50Km away and the mean time of arrival at the hospital was around one hour. The groups were homogeneous (Table 2).

**Table 2:** Descriptive measures for demographic variables, distance, and travel time according to study group, p value – Taubaté, Brazil, May/22 – Sep/22.

Variables	Group	N	Mean ± SD	Median	Min–Max	95% CI	p^*^
Age	CG	24	61.8 ± 10.1	63.5	39–77	[57.5; 65.5]	0.268
	GACA	18	58.8 ± 10.1	58.5	42–73	[54.2; 63.3]	
No. of children	CG	24	2.8 ± 1.7	3	0–8	[2.2; 3.6]	0.266
	GACA	18	2.2 ± 1.4	2	0–5	[1.6; 2.9]	
Distance to	CG	24	41.4 ± 49.2	20	0–141	[24.6; 63.9]	0.446
Taubaté	GACA	18	50.9 ± 47.0	54	0–141	[31.4; 73.8]	
Time for arrival	CG	24	62.5 ± 53.9	40	15–180	[44.7; 88.2]	0.601
at the hospital	GACA	18	66.4 ± 51	47.5	10–180	[47.4; 94.7]	

^*^Wilcoxon-Mann Whitney test.

In both groups, GACA (77.8%; n = 14) and CG (83.3%; n = 20), patients had their partners or children as caregivers.

Patients had adenocarcinoma (59.5%; n = 25) or MM (40.5%; n = 17). In those with adenocarcinoma the main organ affected was the large intestine; 88.0% (n = 22) underwent cancer surgery between 2020 and 2022.

Patients with adenocarcinoma underwent the cycle with BFOL and those with MM the cycle with CYBORD. Regarding the type of CT, two (4.8%) patients received adjuvant treatment and 40 (95.2%) palliative treatment. Comparing the groups, homogeneity was observed only for the protocol (p = 0.088) and type of cancer (p = 0.069), although there were more patients with adenocarcinoma in the CG.

Regarding the time of diagnosis, on average, patients had been under treatment for 18 ± 30.5 months. The mean number of previous CT cycles performed was 3.8 (±2.8), ranging from 1 to 12 cycles, and the number of sessions varied between three and four depending on the CT protocol. When comparing the groups, they were homogeneous for these variables.

As for other health problems, 57.1% (n = 24) were hypertensive, 23.8% (n = 10) were diabetic, and 21.4% (n = 9) were dyslipidemic. The distribution of patients with these health problems was similar between groups (p > 0.05).

### Outcome of the Intervention

In both groups, no patient reported CINV during the CT session. Of the patients, 72.2% (n = 13) of the GACA and 83.3% (n = 20) of the CG took antiemetics in the first session according to the chemotherapy protocol (p = 0.6251).

The occurrence of CINV was similar in both groups (Table 3), as well as the use of antiemetics for nausea: Ondansetron hydrochloride, 16.7% (n = 3) of patients in GACA and 16.7% (n = 4) in CG; Dimenhydrinate, 11.1% (n = 2) of patients in GACA and 4.2% (n = 1) in CG; Metoclopramide hydrochloride, 27.8% (n = 5) of patients in GACA and 16.7% (n = 4) in CG. Patients in both groups used medication for vomiting: Ondansetron hydrochloride, 22.2% (n = 4) of patients in GACA and 8.3% (n = 2) in CG; Dimenhydrinate, 4.2% (n = 1) of patients in the CG; Metoclopramide hydrochloride, 11.1% (n = 2) of patients in GACA and 8.3% (n = 2) in CG. When analyzing the occurrence of CINV in each session, a similar decrease was also observed between the groups until the end of the cycle. It should be noted that the BFOL protocol does not administer Oxaliplatin in the second session and its cycle lasted three weeks, while the CYBORD protocol lasted four weeks.

**Table 3:** Distribution of patients according to study group, occurrence of nausea and vomiting, use of antiemetics and p value – Taubaté, Brazil, May/22-Sep22.

NV	Acupuncture			Control			Total		p
	N	%		N	%		N	%	
Nausea									
Yes	10	47.6		11	52.4		21	100	
No	8	44.4		10	55.6		18	100	0.843
Nausea intensity (CTCAE)									
None	8	44.4		10	55.6		18	100	
Grade I	4	44.4		5	55.6		9	100	
Grade II	5	55.6		4	44.4		9	100	
Grade III	1	33.3		2	66.7		3	100	0.907
Medication for nausea									
Yes	8	53.3		7	46.7		15	100	
No	10	41.8		14	58.2		24	100	0.477
Vomiting									
Yes	5	41.7		7	58.3		12	100	
No	13	48.1		14	51.9		27	100	0.708
Medication for vomiting									
Yes	4	44.4		5	55.6		9	100	
No	14	46.7		18	53.3		30	100	0.907
**Total**	**18**	**46.2**		**21**	**53.8**		**39**	**100**	

In the generalized linear model of mixed effects, there was no statistical difference over the sessions between the levels of nausea severity (CTCAE) and the group (p = 1.000) and between the protocols (p = 0.999). The same was observed for the severity of vomiting (CTCAE) and group (p = 1.000) and between protocols (p = 1.000). When the adverse event occurred, there was a predominance of Grade I (mild) vomiting and Grade II (moderate) vomiting occurred only in two patients in the CG and in one patient in the GACA, both in the BFOL protocol.

As for the use of teas for nausea, this was more frequent in the CG: Chamomile (8.3%; n = 2), Mint (4.2%; n = 1), Lemon Balm (4.2%; n = 1), Fennel (12.5%; n = 3), Boldo (4.2%; n = 1), Green Tea (4.2%; n = 1). Only one GACA patient mentioned the use of ginger tea (5.5%; n = 1). For vomiting, teas were also mentioned in the CG: Chamomile (8.3%; n = 2), Mint (8.3%; n = 2), Lemon Balm (4.2%; n = 1) and Fennel (8.3%; n = 2).

The number and proportion of days with CINV was similar between the two groups (Table 4).

**Table 4:** Descriptive measures for the number of days with nausea and vomiting during treatment and per session according to the group – Taubaté, Brazil, 2022.

Variable	Group	n	Mean	SD	Median	Variation	p*
Nausea-days ratio	Acupuncture	18	16.2	21.5	6.6	0–80	
	Control	21	13.2	21.4	4.8	0–66.7	0.668
Nausea - Total days	Acupuncture	18	3.2	4.0	1.5	0–12	
	Control	21	2.6	4.2	1.0	0–14	0.613
Proportion Days vomiting	Acupuncture	18	5.2	9.9	0	0–33.3	
	Control	21	3.7	7.9	0	0–33.3	0.867
Vomiting Total days	Acupuncture	18	1.0	1.9	0	0–7	
	Control	21	0.7	1.4	0	0–5	0.663

For the type of emesis, no statistically significant difference was observed between the groups for acute vomiting (p = 0.244) and for delayed vomiting (p = 0.966). Delayed emesis was more frequent than acute emesis. When comparing patients by CT protocol, it was observed that there was a higher prevalence of delayed vomiting in patients in the BFOL protocol, despite not using oxaliplatin in the second session.

The analysis by type of protocol also did not show statistically significant differences for the presence of vomiting between groups. No GACA patients experienced vomiting in session four of the CYBORD group. The comparison of the presence of vomiting at home according to each week after the respective session also did not show a statistically significant difference.

## DISCUSSION

In this study, the provision of acupuncture services in an oncology outpatient setting was well received by patients, caregivers, and hospital staff. Recognition of the importance of integrative oncology in the care of cancer patients has been gaining ground among health professionals. The *PICS* carried out in conjunction with chemotherapy, radiotherapy, surgery, and molecular therapy aim at the search for the main role of the patient in their health care process, rescuing bioethical principles and stimulating their autonomy^(16)^.

Regarding the effectiveness of an acupuncture protocol with the systemic points PC6, RN12, SP6, ST36, LR14 and auriculotherapy in the points Shenmen, Spleen, Stomach, Point Zero, Point 29a kinetosis/nausea and Anxiety for the relief of CINV with drugs of high and moderate emetogenic degree, no statistically significant results were obtained in the evaluation made by the Diary of Nausea and Vomiting.

The analysis of the participants’ characteristics showed a prevalence of female patients, with a mean age of around 60 years, white, married, who lived with their partner, who had their partner and/or children as caregivers, and most of them attended the elementary School. This profile was similar to the study on the prevalence of cancer diagnosis in the older people, which showed a mean age of 69.8 years, mostly female, white (71.7%), who lived with their spouse (58.0%), without education or with uncompleted elementary education (62.0%)^(17)^.

Unlike this study, data from the National Cancer Institute show that the incidence of cancer is 17% higher in male patients than in female patients^(18)^.

The National Cancer Policy^(19)^ emphasizes the provision of treatment in a timely manner close to the municipality of origin. In the study, most patients lived outside the city of Taubaté, up to a distance of about 140Km. A study with retrospective cross-sectional analysis examined the data from *SUS* on geographic accessibility to cancer treatment and showed a total of 12,751,728 patients, among which more than half needed to travel from their cities of residence to undergo cancer treatment^(20)^.

The low geographic accessibility to health services corroborates the low use of these services, generating worse health outcome. As cancer treatment is very important and usually involves surgery, chemotherapy and/or radiotherapy, it requires patients to visit the health service several times, and when the distance makes access to these services difficult, treatment is delayed, which may lead these patients to worse prognosis or even death. Travel needs are associated with a more advanced stage of the disease at the time of diagnosis, inadequate treatment, and worse prognosis, affecting quality of life^(21)^.

The focus of the study was patients with solid cancer, such as colorectal (CRC), small intestine and stomach cancer, undergoing chemotherapy with BFOL protocol consisting of drugs such as Oxaliplatin, 5-Fluouracil, and Folinic Acid. Most patients had adenocarcinoma of the large intestine, colon, rectosigmoid or rectum, which is in line with the worldwide cancer prevalence of 90%^(18)^.

Regarding hematological cancer, patients with MM, undergoing chemotherapy treatment with the CYBORD protocol consisting of the drugs Cyclophosphamide, Decadron, Zoledronic Acid and Bortezomib were studied. Epidemiological data from one study observed that the incidence of MM is two to three times higher in black individuals compared to white individuals; however, in the present study, the proportion of MM in white and black patients was almost equal. On average, the patients in the present study had been undergoing treatment for around a year and a half and, half of them had already undergone an autologous stem cell transplant. Studies show that the prevalence of MM has increased due to better diagnostic techniques and improved patient survival, due to autologous hematopoietic stem cell transplantation and the development of new therapeutic agents^(22)^. Even so, in the present study, all patients were undergoing palliative CT.

The comorbidities presented by patients undergoing chemotherapy were hypertension, diabetes, and dyslipidemia. This is different from a study that observed hypertension, as well as a higher prevalence of heart disease, depression and lung disease in older people diagnosed with cancer. Comorbidities can make cancer treatment difficult; however, the implications and management become increasingly important due to population aging and the growing number of older people with cancer^(17)^.

In the present study, an acupuncture protocol was used combining points PC6, RN12, ST36, SP6, LR14 and five acupuncture points obtained from individual assessment by TCM, to relieve CINV. It should be noted that this number of points was higher than that observed in the literature and not all patients used antiemetics during the CT session or between sessions.

The analysis of the nausea and vomiting diary showed that the study groups presented relief of these symptoms in a similar way, both in relation to the occurrence of events and the intensity of nausea and use of antiemetics. Regarding vomiting, despite there being no statistical difference, GACA had less mention of vomiting than the CG, but both groups used antiemetics.

The acupuncture protocol proposed was partially in accordance with the clinical trial protocol that included points PC6, RN12, ST36^(23)^. This randomized clinical trial involved patients with lung cancer, breast cancer, or gynecological cancer receiving chemotherapy with cisplatin, anthracycline, or taxane-based CT regimens; it compared a Sham group to the group treated with points PC6, ST25, LR13, ST36, RN6, and RN12. Treatments were performed for 30 min, twice a day, for four consecutive days. Participants in both groups received intravenous ondansetron as the basis of their antiemetic regimen. However, the effectiveness of this protocol was not satisfactory, with no noticeable improvement in the acupuncture group compared to the control group. Nevertheless, there was significant relief in the severity of nausea (Day 3 and Day 21) and vomiting (Day 7 to Day 21) (p = 0.021). Although there was no significant improvement in vomiting within 120 hours after starting CT, there was improvement in the severity of vomiting in the acupuncture group from the seventh day onwards (p = 0.033)^(23)^.

In a cross-sectional study, 68 patients undergoing chemotherapy treatment with high and moderate emetogenic drugs associated with radiotherapy were randomized into the acupuncture group (point PC6 bilaterally) and the Sham group (75% women, mean age 56 years, 53% with gynecological cancer, 43% CRC, and 4% other types of cancer). An average of 10 sessions were held over four weeks, an average of two sessions per week. Both groups were compared to a reference group receiving only standard antiemetic treatment. There was greater consumption of antiemetics in the Sham group than in the acupuncture group (p = 0.019, RR 1.81, CI 1.06–3.09). Patients treated with acupuncture experienced less intensity of nausea than other patients (p = 0.049). There was a non-significant trend for more patients in the Sham group or reference group to experience more nausea (21 of 31; 68%) than patients receiving acupuncture (9 of 17; 53%: p = 0.074, RR 1.58, CI 0.91– 2.74)^(24)^.

In a Korean study to evaluate the effects of auriculotherapy on nausea, vomiting and retching in patients with CRC in CT, patients were allocated to a CG and an auriculotherapy group. Patients received folinic acid, fluorouracil, and oxaliplatin and the study outcome was assessed by the Korean version of the Nausea, Vomiting and Nausea Index (NVI). The auriculotherapy protocol included the zero points, stomach, brain stem, Shenmen and cardia with prior massaging of these locations to stimulate blood circulation and again a stimulus after placing clay spheres glued into a plaster. Ten auriculotherapy sessions were carried out and patients were instructed to stimulate the points three times a day or whenever they felt nauseated. The auriculotherapy group showed significant relief from nausea, retching, and vomiting compared to the CG. The global NVI score showed a significant difference between groups, with treatment time and interaction with time and groups^(25)^.

The population of the present study also included CRC patients using the same chemotherapy protocol as the literature study^(25)^. Regarding the auriculotherapy protocol, although some points were similar, there was variation in the material fixed to the points, the lack of massage before and after the application of the spheres, and the additional use of five individualized points according to TCM evaluation. There was a tendency towards a reduction in nausea and vomiting, but with no statistical difference in the instruments evaluated.

### Limitation of Study

The limitations of the study were low number of patients in the sample; patient transference to another oncology service; unfeasibility of controlling the use of antiemetics; lack of guidance for patients to use antiemetics only when they have symptoms; two different CT protocols and number of sessions in each cycle; non-use of oxaliplatin in the BFOL cycle (moderate emetogenic potential) in the second session; patients undergoing palliative treatment, who are more energetically weakened and perhaps require longer intervention; failure to assess patients’ perception regarding the use of acupuncture for their health status.

## CONCLUSION

The pilot study was not effective in demonstrating the potential of the proposed protocol in alleviating CINV. The low frequency of CINV and the low number of patients in each chemotherapy protocol compromised the evaluation of the effect of the acupuncture and auriculotherapy protocol in relieving CINV. The reproducibility of the study is suggested, considering the limitations found in choosing an adequate sample of patients.
